# Shared features of metaplasia and the development of adenocarcinoma in the stomach and esophagus

**DOI:** 10.3389/fcell.2023.1151790

**Published:** 2023-03-13

**Authors:** Yongji Zeng, Qing K. Li, Sujayita Roy, Jason C. Mills, Ramon U. Jin

**Affiliations:** ^1^ Section of Gastroenterology, Department of Medicine, Baylor College of Medicine, Houston, TX, United States; ^2^ Department of Pathology, Johns Hopkins Medical Institutions, Baltimore, MD, United States; ^3^ Departments of Medicine, Pathology and Immunology, and Molecular and Cellular Biology, Baylor College of Medicine, Houston, TX, United States; ^4^ Section of Hematology/Oncology, Department of Medicine, Baylor College of Medicine, Houston, TX, United States

**Keywords:** plasticity, paligenosis, Barrett’s esophagus, intestinal metaplasia, pseudopyloric metaplasia, SPEM

## Abstract

**Introduction:** Plasticity is an inherent property of the normal gastrointestinal tract allowing for appropriate response to injury and healing. However, the aberrancy of adaptable responses is also beginning to be recognized as a driver during cancer development and progression. Gastric and esophageal malignancies remain leading causes of cancer-related death globally as there are limited early disease diagnostic tools and paucity of new effective treatments. Gastric and esophageal adenocarcinomas share intestinal metaplasia as a key precancerous precursor lesion.

**Methods:** Here, we utilize an upper GI tract patient-derived tissue microarray that encompasses the sequential development of cancer from normal tissues to illustrate the expression of a set of metaplastic markers.

**Results:** We report that in contrast to gastric intestinal metaplasia, which has traits of both incomplete and complete intestinal metaplasia, Barrett's esophagus (i.e., esophageal intestinal metaplasia) demonstrates hallmarks of incomplete intestinal metaplasia. Specifically, this prevalent incomplete intestinal metaplasia seen in Barrett's esophagus manifests as concurrent development and expression of both gastric and intestinal traits. Additionally, many gastric and esophageal cancers display a loss of or a decrease in these characteristic differentiated cell properties, demonstrating the plasticity of molecular pathways associated with the development of these cancers.

**Discussion:** Further understanding of the commonalities and differences governing the development of upper GI tract intestinal metaplasias and their progression to cancer will lead to improved diagnostic and therapeutic avenues.

## Introduction

Cancers of the stomach and esophagus are major causes of worldwide cancer-related morbidity and mortality with an estimated 1.7 million new cases each year resulting directly in over 1.3 million cancer-related deaths (Sung et al., 2021). In the United States, there were over 48,000 new cases of stomach and esophageal cancers with 27,000 deaths in 2022 (Siegel et al., 2022). In the developed world, adenocarcinoma histologic subtypes predominate and carry dismal 5-year survival rates of only 20%–25% (Rubenstein and Shaheen, 2015; Coleman et al., 2018; Thrift and El-Serag, 2020). While new treatment paradigms are being explored (Zeng and Jin, 2022), current curative treatments remain restricted to a relatively limited arsenal comprised of conventional chemotherapy, chemo-radiotherapy, and surgical resection of the tumor (Bonenkamp et al., 1999; van Hagen et al., 2012; Shapiro et al., 2015; Al-Batran et al., 2019). Thus, patients would benefit from an improved understanding of the pathogenesis of these disease leading to improved early disease detection tools, and safer and more efficacious treatments.

In general, with the exception of tumors arising in the proximal region of the stomach (cardia), gastric and esophageal adenocarcinomas have been considered separate entities in terms of precursor lesions, progression to malignancy, and subsequent clinical course of tumors (Ajani et al., 2017; Smyth et al., 2017; Saenz and Mills, 2018; Singh et al., 2021; Souza and Spechler, 2022). However, accumulating data indicate that gastric and esophageal adenocarcinomas may have considerably more in common than previously appreciated. Genomic profiling has revealed distinct molecular subtype similarities. The ‘Singapore-Duke’ study (Lei et al., 2013), the Asian Cancer Research Group (ACRG) study (Cristescu et al., 2015), and The Cancer Genome Atlas (TCGA) studies (Cancer Genome Atlas Research Network, 2014; Cancer Genome Atlas Research Network, 2017) have provided the basis for the molecular classification of gastroesophageal cancers (Zeng and Jin, 2022). The predominant molecular subtype for gastric adenocarcinomas (over 52%) and esophageal adenocarcinomas (over 98%) is the Chromosomal Instability (CIN) subtype (Cancer Genome Atlas Research Network, 2014; Cancer Genome Atlas Research Network, 2017). Importantly, these CIN gastric and esophageal adenocarcinomas arise in a background of metaplasia and share common intestine-like features. The metaplastic precursor lesion to esophageal adenocarcinoma (EAC) is defined by replacement of the normal squamous epithelial lining of the esophagus with metaplastic columnar cells in response to chronic gastric refluxate (Spechler et al., 2010; Spechler and Souza, 2014). Metaplasia in the body of the stomach is the precursor lesion to gastric adenocarcinoma (GA), and arises during chronic atrophic gastritis through epithelial reprogramming to a more distal antral-like state (Goldenring and Mills, 2022). Such pyloric or pseudopyloric metaplasia occurs in response to chronic damage induced predominantly by *Helicobactor pylori* infection (Correa, 1992; Goldenring and Mills, 2022). In both the esophagus and stomach, metaplasia can assume an intestine-like state. In the esophagus, such metaplasia is called Barrett’s esophagus (BE), and in the stomach it is known as gastric intestinal metaplasia (GIM).

Here, we performed an immunohistologic examination to compare metaplasia and oncogenesis in the esophagus and stomach. We used a set of markers normally restricted to specific regions of the gastrointestinal epithelium whose expression is induced in metaplasia. We immunostained a microarray of human tissue exhibiting progressive phases of oncogenesis in the esophagus and stomach using antibodies against: trefoil peptides (TFF2 and TFF3), mucins (MUC2, MUC5AC, and MUC6), and transcription factors (CDX2 and SOX2). Our results indicate a similarity between BE and incomplete GIM. By definition, incomplete GIM manifests as the concomitant expression of both gastric and intestinal markers, which we also observe in BE. The mixed gastric and intestinal differentiated cell features seen in BE and GIM are apparently decreased or lost during oncogenesis. Our sampling of malignant cells largely showed decreased expression of metaplasia markers, suggesting the plasticity of molecular pathways involved in cancer development. Further elucidation of the similarities we identify here in the processes governing the development of metaplasia and cancer progression in the esophagus and stomach could lead to improved diagnostic and therapeutic avenues.

## Materials and methods

### Study case collection

Clinical cases included in our study were collected between January 2011 and December 2018 at Johns Hopkins Hospitals with patient informed consent. All tissue samples were obtained by esophagogastroduodenoscopy (EGD) biopsy, surgical resection, and/or endoscopic mucosal resection (EMR) procedures. Relevant clinical and pathological information for study cases was reviewed and included in the study. The dysplasia grade for Barrett’s esophagus cases was determined according to the American College of Gastroenterology (ACG) 2022 guidelines (Shaheen et al., 2022) and the WHO classification of digestive system tumors (Nagtegaal et al., 2020). The pathological stage of resected carcinomas was determined according to the eighth edition of the American Joint Committee on Cancer (AJCC) Cancer Staging Manual (Amin et al., 2017) and the WHO classification of digestive system tumors (Nagtegaal et al., 2020). All pathological diagnoses were determined by pathologists certified by the American Board of Pathology and reviewed by gastroenterology pathologists.

The use of pathology material for the study was approved by the Institutional Review Board of Johns Hopkins Medical Institutions. In addition, all methods performed in the study were in accordance with the relevant guidelines and regulations.

### Construction of the tissue microarrays

The tissue microarrays (TMA) were constructed using the above-mentioned clinical biopsy, surgical resection, and/or endoscopic mucosal resection tissue samples. All tissues were fixed in 10% formalin and embedded in paraffin. To verify the original pathology reports, hematoxylin and eosin (H&E) stained sections were re-reviewed by a pathologist certified by the American Board of Pathology (QKL) prior to TMA construction to ensure the accurate representation of the lesional area and matched normal tissue. Furthermore, H&E stained slides of individual cases were reviewed, the best representative slides were selected for each case, and the corresponding tissue blocks were selected for TMA inclusion. TMA cores (at a diameter of 2.0 mm for BE cases, and 1.0 mm for all other cases) were obtained from paraffin blocks and transferred to build the TMA blocks. In our TMAs, 3-5 cores were obtained from single tissue blocks, except in two cases of BE due to scant biopsy material, in which case two cores were obtained from each block. After the construction of the TMAs, H&E stained TMA slides were re-reviewed by a pathologist (QKL) as well as by a pathologist specializing in esophageal and gastric oncogenesis (JCM) to ensure the pathological diagnosis of individual cores.

### Immunohistochemical staining of markers

The immunohistochemical (IHC) assays were performed by the Oncology Tissue Services Core at The Johns Hopkins University School of Medicine. Briefly, the TMA blocks were cut into 4 µm unstained slides. All unstained slides were deparaffinized prior to incubation with primary antibodies. Following dewaxing and rehydration, epitope retrieval was performed using Ventana Ultra CC1 buffer (catalog# 6414575001, Roche Diagnostics) at 96°C for 64 min. All IHC stains were performed on a Ventana Discovery Ultra autostainer (Roche Diagnostics). Primary antibodies were applied at 36°C for 60 min, and then detected using an anti-mouse or anti-rabbit HQ detection system (catalog# 7017936001 or 7017782001, Roche Diagnostics) followed by Chromomap DAB IHC detection kit (catalog # 5266645001, Roche Diagnostics), counterstaining with Mayer’s H&E, dehydration, and mounting. For SOX2 and CDX2 IHC stains, the counterstaining step was performed with eosin only. Appropriate positive and negative controls were included in the staining assays. The complete details of primary antibodies are summarized in Table 1.

**TABLE 1:** Summary of primary antibodies.

Antibody	Company	Clone	Clonality	Dilution	Catalog #
TFF2	Sigma-Aldrich	N/A	Rabbit Polyclonal	1:250 (0.1 mg/mL)	HPA036705
TFF3	Sigma-Aldrich	N/A	Rabbit Polyclonal	1:2000 (0.1 mg/mL)	HPA035464
MUC2	Leica	Ccp58	Mouse Monoclonal	Prediluted	PA0155
MUC5AC	Cell Marque	MRQ-19	Mouse Monoclonal	1:2000	292M-94
MUC6	Abcam	MUC6/916	Mouse Monoclonal	1:200	Ab216017
CDX2	Abcam	EPR2764Y	Rabbit Monoclonal	1:2000 (0.866 mg/mL)	Ab76541
SOX2	Santa Cruz	E-4	Mouse Monoclonal	1:100 (200 ug/mL)	Sc-365823

### Evaluation and scoring of IHC staining

The strength of staining for each marker (taking into account membranous, cytoplasmic and nuclear staining patterns) was scored by the pathologist (QKL) using a semi-quantitative four-tier system: score 0 (0% of cells stained, no staining), score 1 (<10% of cells stained, weak and sparse focal staining), score 2 (10%–50% of cells stained, medium and focal staining), or score 3 (>50% of cells stained, strong and diffuse staining) (Ao et al., 2014; Gniadek et al., 2017; Zhu et al., 2019; Jin et al., 2021; Li et al., 2021; Johnson et al., 2022; Li et al., 2022). Care was taken not to interpret entrapped macrophages or mucinous material as positive staining.

All IHC stained slides were scanned using the Concentriq Digital Pathology Platform (Proscia) and stored as digital files. Each core was considered as an individual data point, as more than one core might be obtained from the same tissue block described in detail above (Ao et al., 2014; Gniadek et al., 2017; Zhu et al., 2019; Jin et al., 2021; Li et al., 2021; Johnson et al., 2022; Li et al., 2022). Depending on the TMA section, not all cores could be evaluated due to the loss of tissue during processing or sectioning.

### Data analyses and statistics

For each phenotype, the average histological score was calculated by averaging all the core staining scores for the corresponding diagnostic phenotype, and results were plotted using GraphPad Prism 9.0. In addition, the relative fraction of tissue cores with each score was analyzed by SPSS 19.0 (IBM) for each phenotype, and results were plotted using GraphPad Prism 9.0. The differential expression of individual markers among phenotypic groups was analyzed by the Fisher’s exact test (Supplementary Tables S1, S2). All tests were two-sided with a *p*-value <0.05 considered statistically significant.

## Results

### Demographics of the TMA study cases

The tissue microarray patient demographics are summarized in Table 2. Overall, we assessed a total of 40 cases from the esophagus and 12 cases from the stomach. As mentioned above, multiple TMA cores may have been taken from a single case based on the presence or absence of specific diagnoses in the sampled region as determined by our expert pathologists. The patients included in our study were mostly males of the White/Caucasian race/ethnicity with an average age of 65 years old.

**TABLE 2:** Demographics of the study cases.

Characteristic		No.	
Location		Esophagus^a^(n = 40)	Stomach^a^(n = 12)
Sex	Female,n(%)	5(12.5)	2(16.7)
	Male,n(%)	35(87.5)	10(83.3)
Race/Ethnicity	Hispanic or Latino,n(%)	0(0.0)	1(8.3)
	African American,n(%)	1(2.5)	1(8.3)
	Asian,n(%)	0(0.0)	1(8.3)
	White/Caucasian,n(%)	35(87.5)	9(75.0)
	Other^b^,n(%)	4(10.0)	0(0.0)
Age, years (mean [range])		65.5[50–90]	64.5[49–76]
	≥60 years,n(%)	32(80.0)	8(66.7)
	<60 years,n(%)	8(20.0)	4(33.3)
Procedure	Biopsy,n(%)	22(55.0)	2(16.7)
	Endoscopic Mucosal Resection,n(%)	2(5.0)	0(0.0)
	Surgical Resection,n(%)	16(40.0)	10(83.3)
Diagnosis	Intestinal Metaplasia,n(%)	N/A^c^	6(50.0)
	Adenocarcinoma,n(%)	30(75.0)	11(91.7)
	with Lymph Node Metastasis,n(%)	5(12.5)	5(41.7)
	with Liver Metastasis,n(%)	0(0.0)	2(16.7)
	Barrett’s Esophagus		
	with Low-Grade Dysplasia,n(%)	13(32.5)	N/A
	with High-Grade Dysplasia,n(%)	10(25.0)	N/A
	Negative for Dysplasia,n(%)	15(37.5)	N/A
Proton Pump Inhibitors Treatment	Treatment,n(%)	23(57.5)	9(75.0)
	No treatment,n(%)	17(42.5)	3(25.0)

^a^ In our study, three patients had both a biopsy and follow-up stomach cancer surgical resection; we considered these cases separately.

^b^ Including Native American or Alaska Native, Native Hawaiian or Pacific Islander, or other.

^c^ Cases of esophageal intestinal metaplasia were classified as Barrett’s esophagus.

In our cohort, we examined the presence of intestinal metaplasia, dysplasia and adenocarcinoma. A total of 38 cases of Barrett’s esophagus (15 negative for dysplasia, 13 with low-grade dysplasia, and 10 with high-grade dysplasia) and 6 cases of GIM were analyzed. In addition, 30 EACs and 11 GAs cases were included. A subset of our study cases, including 15 EACs and 11 GAs were treated with curative surgical or endoscopic mucosal resection. At time of resection, five of the EACs had lymph node metastases, while five and two of the GAs had lymph node and liver metastases, respectively. Many of these resected cancer cases had received neoadjuvant chemoradiation or chemotherapy prior to surgery, including 11 of 15 EACs and 5 of the 11 GAs. Our tissue microarray demonstrates an inclusive clinical representation of normal, metaplasia, and adenocarcinoma cases for the esophagus and stomach based on patient demographics.

### A tissue microarray that encompasses sequential formation of esophageal and gastric adenocarcinomas

**FIGURE 1:**
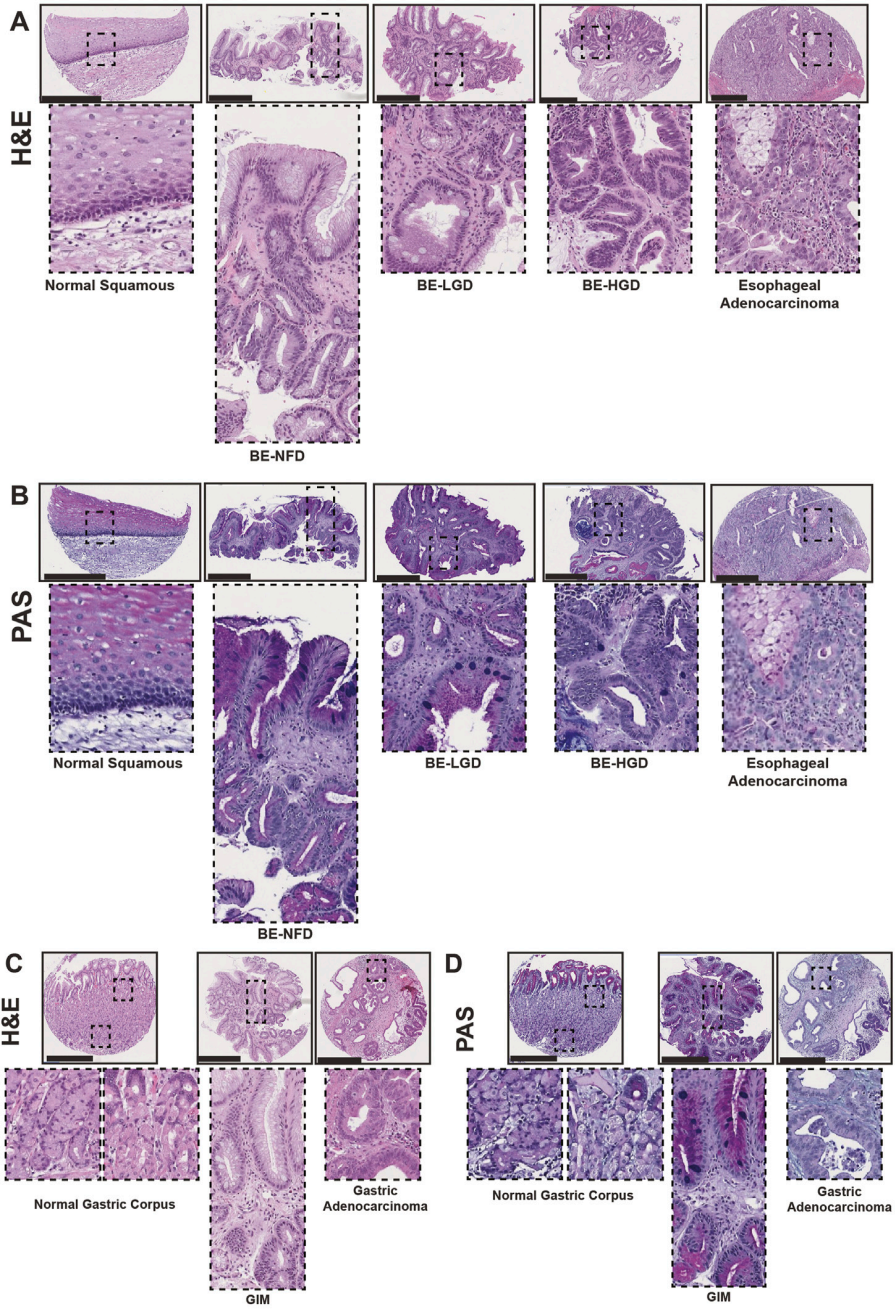
Human tissue microarray of normal squamous-BE-EAC and normal gastric gland-GIM-GA progression sequence. **(A)** Morphology of representative H&E staining from the human tissue microarray of esophageal cases, from left to right: normal squamous, BE-NFD, BE-LGD, BE-HGD, and EAC. **(B)** Morphology of representative PAS staining from the human tissue microarray of esophageal cases, same sequence as in **(A)**. **(C)** Morphology of representative H&E staining from the human tissue microarray of gastric cases, from left to right: normal gastric corpus (left inset from base of the glands, right inset from the surface of the glands), GIM, and GA. **(D)** Morphology of representative PAS staining from the human tissue microarray of gastric cases, in the same sequence as in **(C)**. All scale bars, 500 μm.

H&E and corresponding Periodic Acid–Schiff (PAS) staining showed the histological characteristics of normal squamous, Barrett’s esophagus negative for dysplasia (BE-NFD), Barrett’s esophagus with low-grade dysplasia (BE-LGD), Barrett’s esophagus with high-grade dysplasia (BE-HGD), and EAC from the esophageal cases (Figures 1A, B); and normal gastric corpus, GIM, and GA from the gastric cases (Figures 1C, D). As expected, PAS staining highlighted mucus-containing goblet cells in cores from both BE and GIM samples. In addition, we carefully examined full glands encompassing the top (closest to the lumen of the esophagus or stomach) and the deeper gland areas for BE-NFD and GIM. We did this, as molecular analyses of BE in recent years have highlighted basal, deep glandular mucous cell features in these lesions (Busslinger et al., 2021; Evans et al., 2022). Importantly, we performed all immunohistologic analyses on the same cores using serial sections to demonstrate the relationship of multiple assessed markers on the same tissue areas. From the TMA esophageal and stomach cases, we have also included samples of columnar-lined mucosa of the esophagus (DeMeester and DeMeester, 2000) and complete GIM of the stomach (Correa et al., 2010; Goldenring and Mills, 2022) (Supplementary Figures S1, S2). Our TMAs provide a valuable tool to compare and contrast critical features during the development of BE and GIM and their sequential progression to cancers in the esophagus and stomach.

### TFF2 and TTF3 expression in normal squamous-BE-EAC and normal gastric gland-GIM-GA progression sequences

Pyloric or pseudopyloric metaplasia is a gastric repair process in response to injury usually caused by *Helicobacter pylori* infection (Goldenring et al., 2010; Goldenring and Mills, 2022). During this process, the normal gastric glands exhibit loss of parietal cells, and mature chief cells become metaplastic with abnormal expression of the gastric gland neck cell lineage marker, Trefoil Factor 2 (TFF2) (Weis et al., 2013; Saenz and Mills, 2018; Willet et al., 2018). We note that such metaplastic cells are called spasmolytic polypeptide-expressing metaplasia (SPEM) because TFF2 is now the official name for spasmolytic polypeptide. GIM and gastric cancer are thought to arise from pyloric metaplasia (Goldenring et al., 2010; Graham et al., 2019; Goldenring and Mills, 2022). Also, it should be noted that the lesion “chronic atrophic gastritis” is essentially indistinguishable from pyloric metaplasia as the loss of parietal cells and reprogramming of chief cells are the atrophic changes that give that lesion its name (Goldenring and Mills, 2022; Hsieh et al., 2022).

**FIGURE 2:**
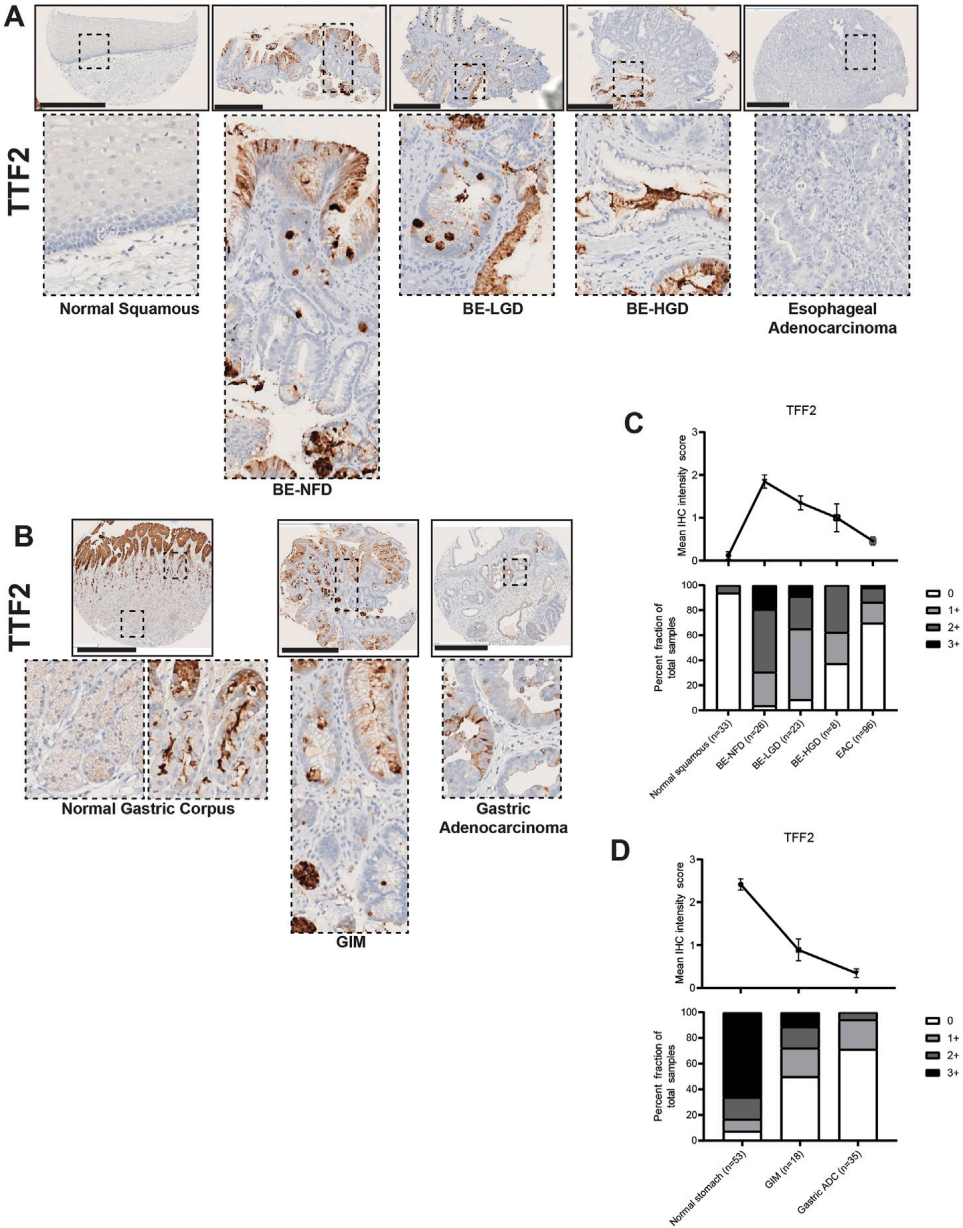
TFF2 expression in normal squamous-BE-EAC and normal gastric gland-GIM-GA progression sequence. **(A)** Immunohistochemistry of TFF2 staining in human tissue microarray esophageal cases, from left to right: normal squamous, BE-NFD, BE-LGD, BE-HGD, and EAC. **(B)** Immunohistochemistry of TFF2 staining in human tissue microarray gastric cases, from left to right: normal gastric corpus (left inset from base of the glands, right inset from the surface of the glands), GIM, and GA. All scale bars, 500 μm. **(C)** Analysis of the human esophageal tissue microarray with normal squamous, BE-NFD, BE-LGD, BE-HGD, and EAC tissue cores stained by immunohistochemistry for TFF2. Top: the TFF2 average IHC intensity score of each esophageal phenotype is plotted. For staining intensity, score 0 (undetectable) to 3 (most intense). Bottom: the TFF2 fraction of esophageal tissue cores with each score is plotted. Each phenotype’s total tissue core number is provided at the bottom of each column. **(D)** Analysis of the human gastric tissue microarray with normal, GIM, and GA tissue cores stained by immunohistochemistry for TFF2. Top: the TFF2 average IHC intensity score of each gastric phenotype is plotted. For staining intensity, score 0 (undetectable) to 3 (most intense). Bottom: the TFF2 fraction of gastric tissue cores with each score is plotted. Each phenotype’s total tissue core number is provided at the bottom of each column.

In our study, TFF2 was expressed in intestinal metaplasia lesions in both the esophagus and stomach (Figures 2A, B). As expected, TFF2 was not expressed in the normal esophageal squamous epithelium, but its expression was correlated with the development of BE (Figure 2A **)**. In the normal gastric corpus, TFF2 was expressed in mucous neck cells with no expression in mature chief cells at the gland bases (Figure 2B). We also detected TFF2 in foveolar pit cells, which may be an inadvertent cross-reaction against TFF1 as the two antigens are over 60% homologous, and similar pit-cell TFF2 reactivity has been reported before (Hoffmann, 2015; Hsieh et al., 2022). Given that TFF2 is a gastric marker, it was predictably decreased in GIM (Figure 2B). However, TFF2 was abundantly detected in BE with foci of expression at the surface and more intense positivity in deep glandular cells. Interestingly, TFF2 expression levels decreased with the development of dysplastic BE and EAC (Figure 2C; Supplementary Table S1). There was a similar TFF2 decreasing expression pattern with the development of GA (Figure 2D; Supplementary Table S2).

**FIGURE 3:**
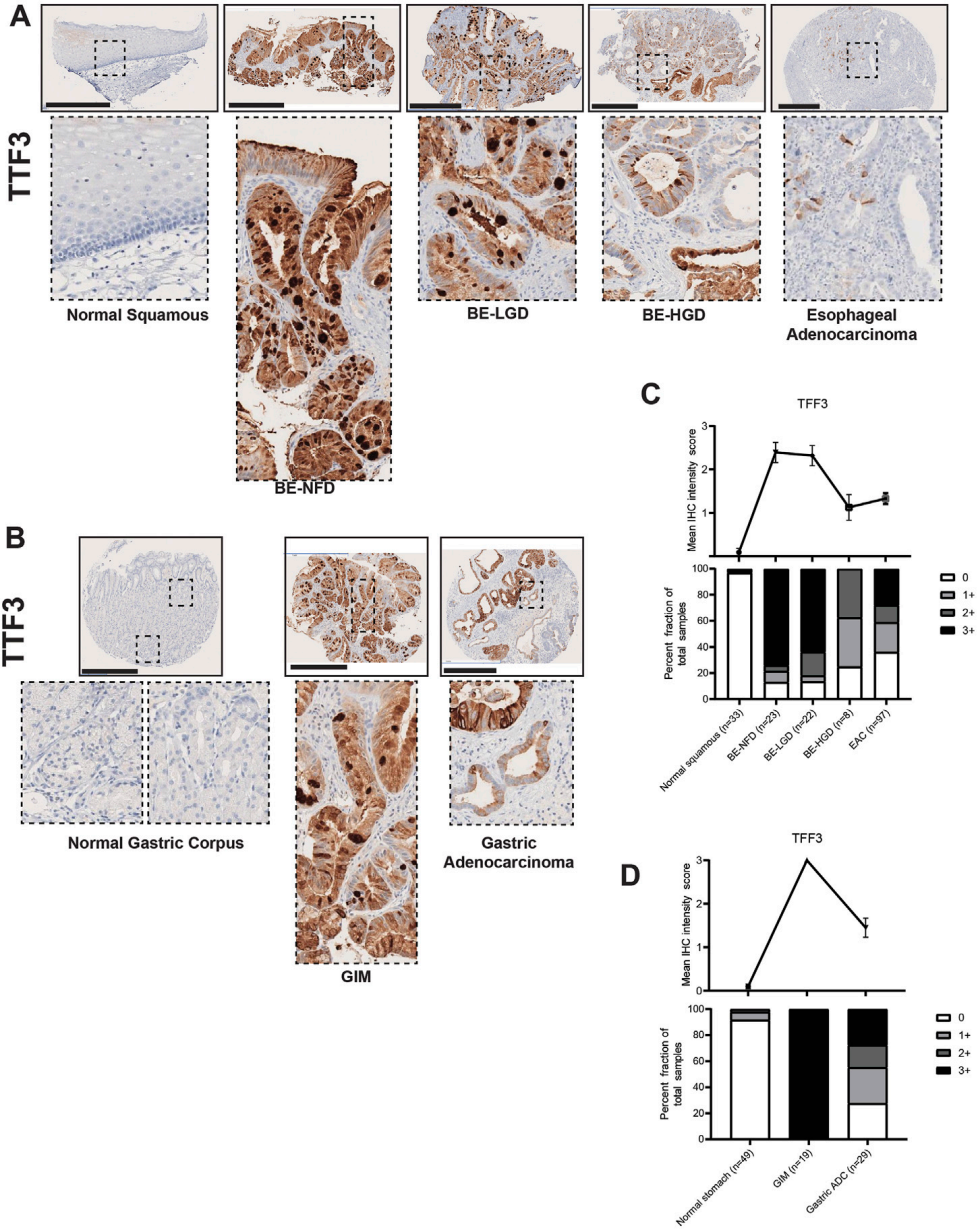
TFF3 expression in normal squamous-BE-EAC and normal gastric gland-GIM-GA progression sequence. **(A)** Immunohistochemistry of TFF3 staining in human tissue microarray esophageal cases, in the same sequence as in Figure 2A. **(B)** Immunohistochemistry of TFF3 staining in human tissue microarray gastric cases, in the same sequence as in Figure 2B. All scale bars, 500 μm. **(C)** Top: the TFF3 average IHC intensity score of each esophageal phenotype is plotted in the same sequence as in Figure 2C. Bottom: the TFF3 fraction of esophageal tissue cores with each score is plotted in the same sequence as in Figure 2C. Each phenotype’s total tissue core number is provided at the bottom of each column. **(D)** Top: the TFF3 average IHC intensity score of each esophageal phenotype is plotted in the same sequence as in Figure 2D. Bottom: the TFF3 fraction of esophageal tissue cores with each score is plotted in the same sequence as in Figure 2D. Each phenotype’s total tissue core number is provided at the bottom of each column.

Expression of another trefoil factor protein, Trefoil Factor 3 (TFF3), has been shown to be increased in BE (Lauren, 1965; Lao-Sirieix et al., 2009; Fitzgerald et al., 2014; Lavery et al., 2014; Fitzgerald et al., 2020) and also strongly induced during gastric mucosal injury (Taupin et al., 2001). Accordingly, our TMA immunohistochemistry results demonstrated that TFF3 was not significantly expressed in the normal esophagus nor in the normal gastric corpus, but its expression was significantly increased with the development of intestinal metaplasia (Figures 3A–D; Supplementary Tables S1, S2). Furthermore, compared with TFF2 expression in the IM glands, TFF3 was expressed throughout the entire metaplastic gland in a diffuse distribution (Figures 3A, B). We also found that the expression level of TFF3 gradually decreased during the BE metaplasia-to-dysplasia to EAC progression (Figures 3A, C; Supplementary Table S1) and during the GIM to GA progression (Figures 3B, D; Supplementary Table S2). In our study, the samples of columnar-lined mucosa of the esophagus showed dual expression of TFF2 and TFF3 (Supplementary Figure S1), while complete GIM showed predominantly singular expression of TFF3 (Supplementary Figure S2).

In summary, we show that TFF2, which is normally present in the stomach, becomes abundantly expressed in BE, is decreased in expression in GIM, and its expression decreases during gastroesophageal cancer formation. TFF3 is an intestinal marker not expressed normally in the upper GI tract, but is strongly expressed in both BE and GIM, and its expression also decreases during cancer development.

### MUC2, MUC5AC, and MUC6 expression in normal squamous-BE-EAC and normal gastric gland-GIM-GA progression sequences

**FIGURE 4:**
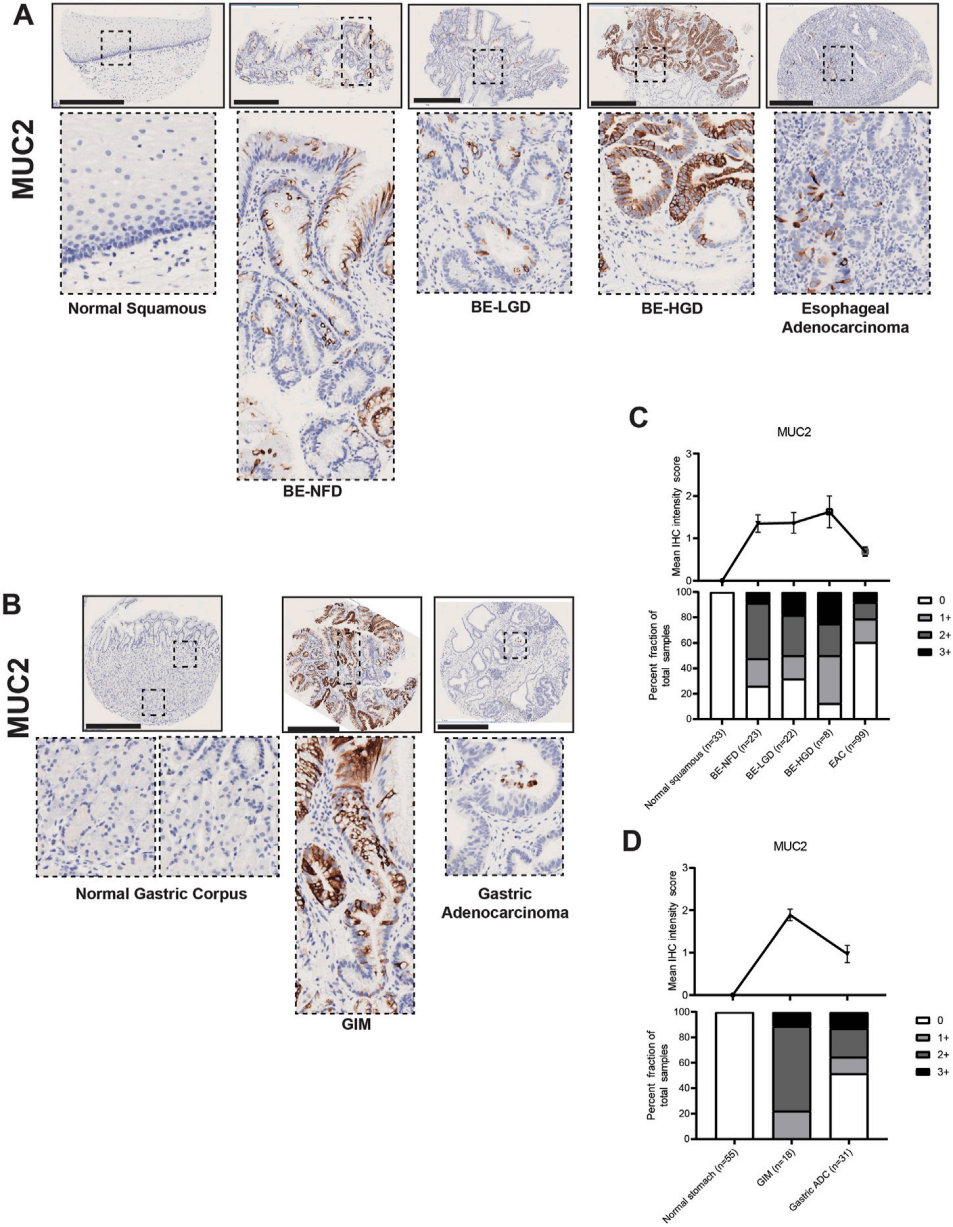
MUC2 expression in normal squamous-BE-EAC and normal gastric gland-GIM-GA progression sequence. **(A)** Immunohistochemistry of MUC2 staining in human tissue microarray esophageal cases, in the same sequence as in Figure 2A. **(B)** Immunohistochemistry of MUC2 staining in human tissue microarray gastric cases, in the same sequence as in Figure 2B. All scale bars, 500 μm. **(C)** Top: the MUC2 average IHC intensity score of each esophageal phenotype is plotted in the same sequence as in Figure 2C. Bottom: the MUC2 fraction of esophageal tissue cores with each score is plotted in the same sequence as in Figure 2C. Each phenotype’s total tissue core number is provided at the bottom of each column. **(D)** Top: the MUC2 average IHC intensity score of each esophageal phenotype is plotted in the same sequence as in Figure 2D. Bottom: the MUC2 fraction of esophageal tissue cores with each score is plotted in the same sequence as in Figure 2D. Each phenotype’s total tissue core number is provided at the bottom of each column.

Mucins are glycosylated proteins expressed throughout the GI tract. MUC5AC is normally expressed in the gastric foveolar pit cells, and MUC6 in gastric mucous neck cells, while MUC2 is not expressed in the normal gastric mucosa, but is rather an intestinal mucin (Reis et al., 1999). Our TMA IHC results showed that both BE and GIM contained MUC2-positive cells (Figures 4A, B). Compared to normal esophageal squamous and normal gastric corpus cells, MUC2 was highly expressed in intestine-specific goblet cells, which were mainly distributed more towards the luminal surface rather than deep within glands in both BE and GIM (Figures 4A, B). In addition, MUC2 expression decreased during progression to EAC and GA (Figures 4A–D; Supplementary Tables S1, S2).

**FIGURE 5:**
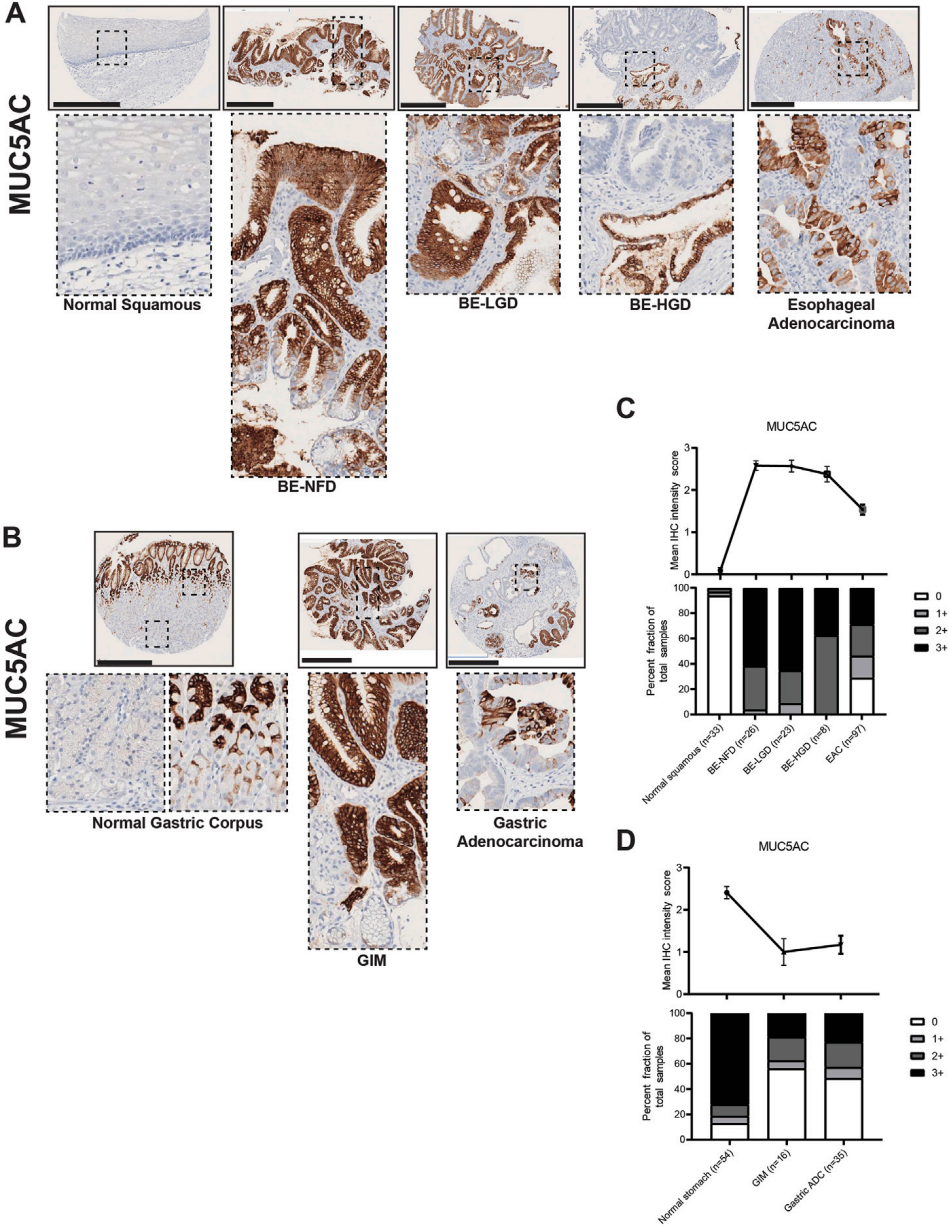
MUC5AC expression in normal squamous-BE-EAC and normal gastric gland-GIM-GA progression sequence. **(A)** Immunohistochemistry of MUC5AC staining in human tissue microarray esophageal cases, in the same sequence as in Figure 2A. **(B)** Immunohistochemistry of MUC5AC staining in human tissue microarray gastric cases, in the same sequence as in Figure 2B. All scale bars, 500 μm. **(C)** Top: the MUC5AC average IHC intensity score of each esophageal phenotype is plotted in the same sequence as in Figure 2C. Bottom: the MUC5AC fraction of esophageal tissue cores with each score is plotted in the same sequence as in Figure 2C. Each phenotype’s total tissue core number is provided at the bottom of each column. **(D)** Top: the MUC5AC average IHC intensity score of each esophageal phenotype is plotted in the same sequence as in Figure 2D. Bottom: the MUC5AC fraction of esophageal tissue cores with each score is plotted in the same sequence as in Figure 2D. Each phenotype’s total tissue core number is provided at the bottom of each column.

Our IHC data demonstrated that MUC5AC was expressed by surface foveolar pit cells in the normal gastric corpus, and mainly distributed in the superficial glandular areas in both BE and GIM (Figures 5A, B). Interestingly, although MUC5AC paralleled the decreasing expression pattern of MUC2 in the BE-NFD, BE-LGD, BE-HGD and EAC progression, MUC5AC expression was decreased in GIM and did not further decrease in GA in our study (Figures 5C, D; Supplementary Tables S1, S2).

**FIGURE 6:**
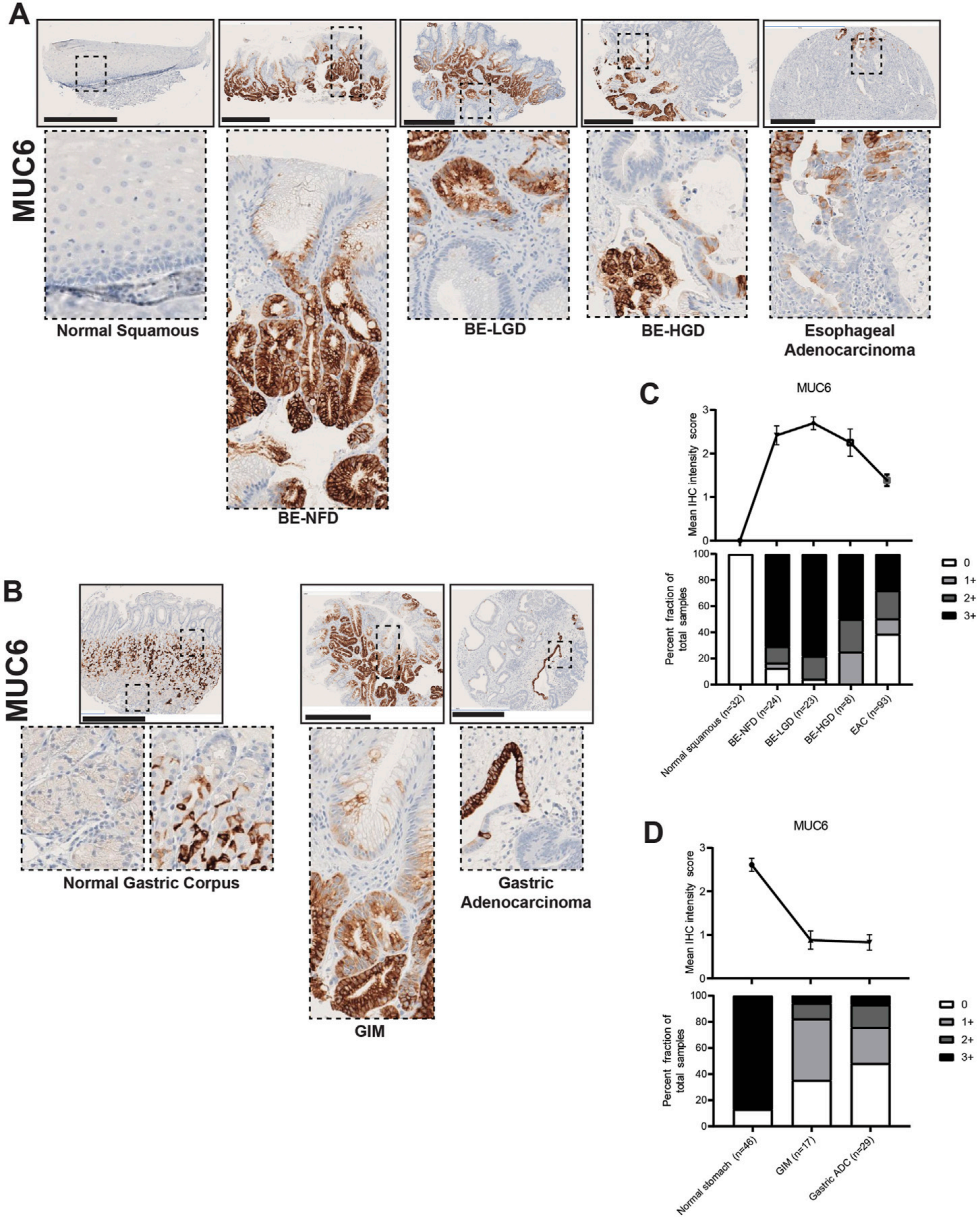
MUC6 expression in normal squamous-BE-EAC and normal gastric gland-GIM-GA progression sequence. **(A)** Immunohistochemistry of MUC6 staining in human tissue microarray esophageal cases, in the same sequence as in Figure 2A. **(B)** Immunohistochemistry of MUC6 staining in human tissue microarray gastric cases, in the same sequence as in Figure 2B. All scale bars, 500 μm. **(C)** Top: the MUC6 average IHC intensity score of each esophageal phenotype is plotted in the same sequence as in Figure 2C. Bottom: the MUC6 fraction of esophageal tissue cores with each score is plotted in the same sequence as in Figure 2C. Each phenotype’s total tissue core number is provided at the bottom of each column. **(D)** Top: the MUC6 average IHC intensity score of each esophageal phenotype is plotted in the same sequence as in Figure 2D. Bottom: the MUC6 fraction of esophageal tissue cores with each score is plotted in the same sequence as in Figure 2D. Each phenotype’s total tissue core number is provided at the bottom of each column.

In contrast to MUC2 and MUC5AC, MUC6 was mainly expressed in the deeper gland regions of BE and GIM (Figures 6A, B). MUC6 was not expressed in the normal squamous epithelium of the esophagus but was expressed in the mucus neck cells of the gastric corpus (Figures 6A, B). Akin to MUC5AC, MUC6 expression decreased with cancer formation in the esophagus but not in the stomach (Figures 6C, D; Supplementary Tables S1, S2). In addition, samples of columnar-lined mucosa of the esophagus showed focal expression of MUC5AC and diffuse expression of MUC6 (Supplementary Figure S1). Complete GIM showed a predominantly singular expression of MUC2 (Supplementary Figure S2).

Here, we show that MUC2, an intestinal marker not expressed in the esophagus or stomach, is strongly expressed in both BE and GIM, and its expression decreases during cancer development. MUC5AC and MUC6, which are normal mucins expressed in the stomach, become aberrantly expressed in the esophagus in nearly all BE lesions. MUC5AC and MUC6 expression is decreased in GIM indicating that many of these lesions (i.e., complete GIM) may lose gastric characteristics. MUC5AC and MUC6 expression decreases during esophageal adenocarcinoma formation, but not gastric adenocarcinoma progression.

### CDX2 and SOX2 expression in normal squamous-BE-EAC and normal gastric gland-GIM-GA progression sequences

**FIGURE 7:**
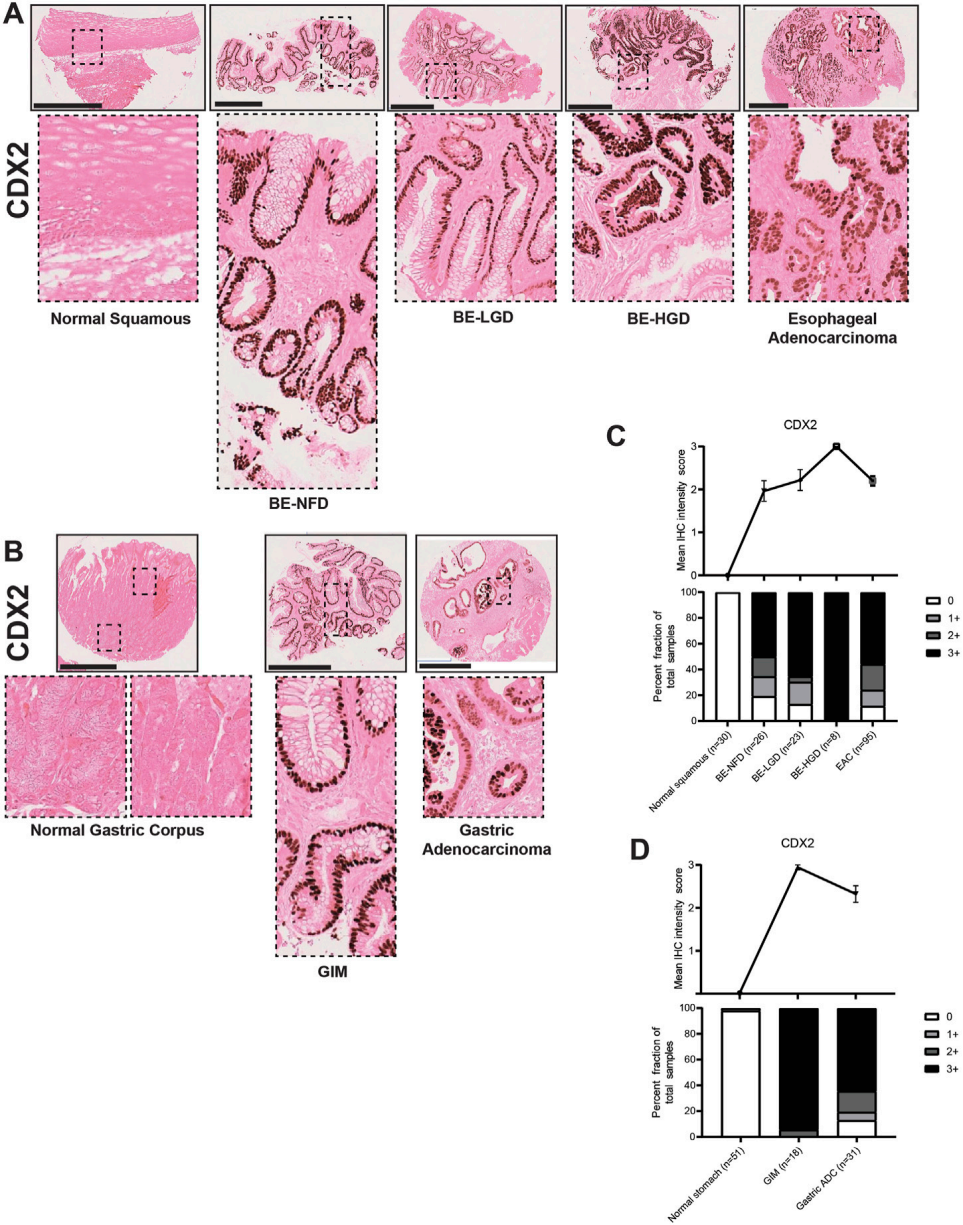
CDX2 expression in normal squamous-BE-EAC and normal gastric gland-GIM-GA progression sequence. **(A)** Immunohistochemistry of CDX2 staining in human tissue microarray esophageal cases, in the same sequence as in Figure 2A. **(B)** Immunohistochemistry of CDX2 staining in human tissue microarray gastric cases, in the same sequence as in Figure 2B. All scale bars, 500 μm. **(C)** Top: the CDX2 average IHC intensity score of each esophageal phenotype is plotted in the same sequence as in Figure 2C. Bottom: the CDX2 fraction of esophageal tissue cores with each score is plotted in the same sequence as in Figure 2C. Each phenotype’s total tissue core number is provided at the bottom of each column. **(D)** Top: the CDX2 average IHC intensity score of each esophageal phenotype is plotted in the same sequence as in Figure 2D. Bottom: the CDX2 fraction of esophageal tissue cores with each score is plotted in the same sequence as in Figure 2D. Each phenotype’s total tissue core number is provided at the bottom of each column.

CDX2 and SOX2 are key transcription factors involved in gut development (McGrath and Wells, 2015; Miao et al., 2020; Ma et al., 2022) with SOX2 involved in foregut development (Que et al., 2007) and CDX2 functioning in intestinal development (Silberg et al., 2000). In our study, CDX2, as expected, had no expression in the normal esophageal squamous epithelium or the normal gastric corpus (Figures 7A, B). However, CDX2 became dramatically expressed in BE, and its expression was increased with the development of dysplasia; CDX2 expression was downregulated but still maintained at high levels in EAC (Figures 7A–C; Supplementary Table S1). Similar to the esophagus, nuclear CDX2 expression was significantly increased in GIM cells, while downregulated and kept at high levels in GA (Figures 7B, D; Supplementary Table S2).

**FIGURE 8:**
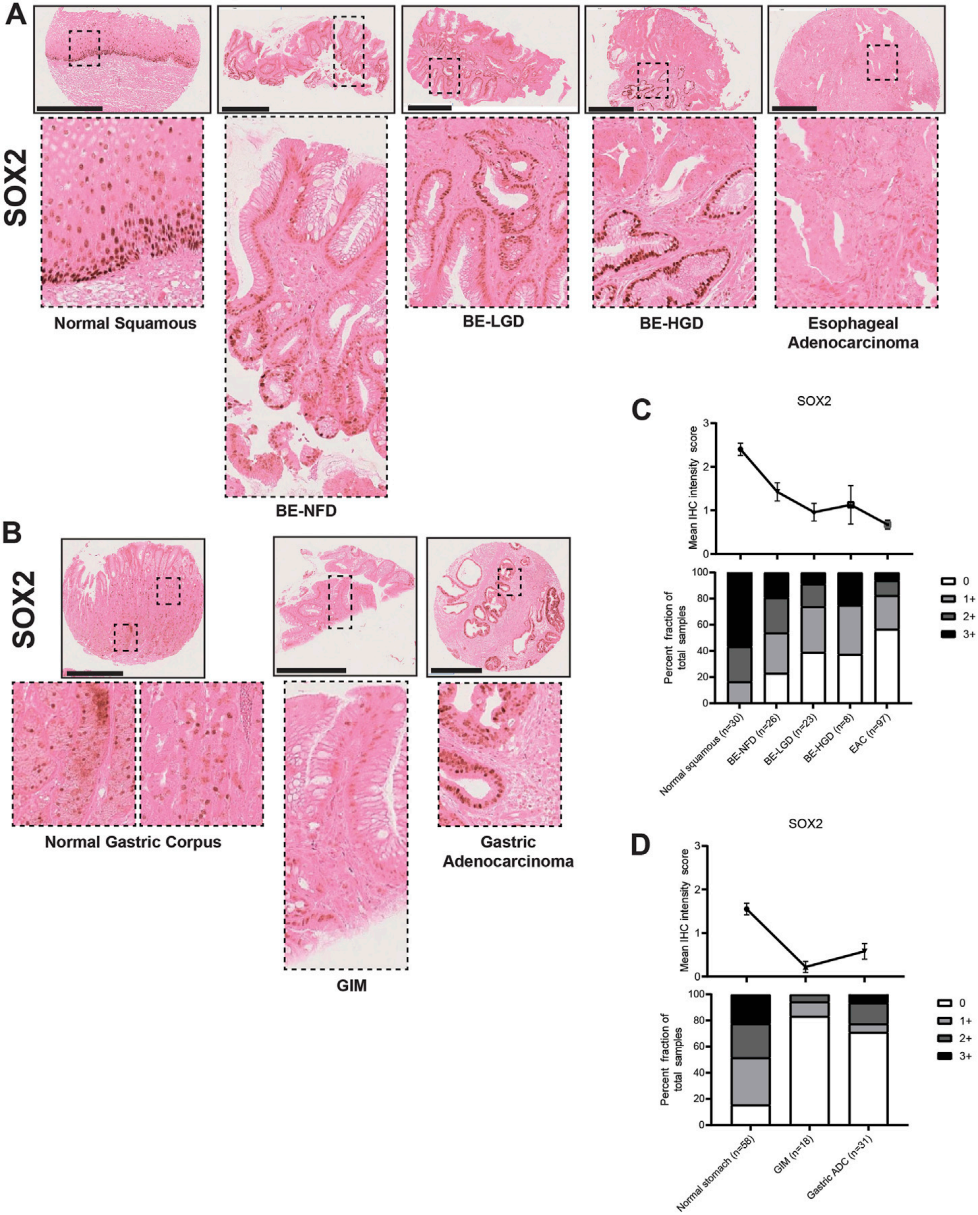
SOX2 expression in normal squamous-BE-EAC and normal gastric gland-GIM-GA progression sequence. **(A)** Immunohistochemistry of SOX2 staining in human tissue microarray esophageal cases, in the same sequence as in Figure 2A. **(B)** Immunohistochemistry of SOX2 staining in human tissue microarray gastric cases, in the same sequence as in Figure 2B. All scale bars, 500 μm. **(C)** Top: the SOX2 average IHC intensity score of each esophageal phenotype is plotted in the same sequence as in Figure 2C. Bottom: the SOX2 fraction of esophageal tissue cores with each score is plotted in the same sequence as in Figure 2C. Each phenotype’s total tissue core number is provided at the bottom of each column. **(D)** Top: the SOX2 average IHC intensity score of each esophageal phenotype is plotted in the same sequence as in Figure 2D. Bottom: the SOX2 fraction of esophageal tissue cores with each score is plotted in the same sequence as in Figure 2D. Each phenotype’s total tissue core number is provided at the bottom of each column.

In the esophagus, SOX2 expression levels were gradually downregulated during normal esophageal squamous, BE metaplasia, dysplasia to EAC progression sequences (Figures 8A, C; Supplementary Table S1). In the stomach, SOX2 was expressed in the normal gastric glands in a predominantly chief cell distribution and decreased in GIM and GA (Figures 8B, D; Supplementary Table S2). The CDX2 and SOX2 expression demonstrated a converse pattern in the metaplasia-dysplasia-adenocarcinoma progression sequence in both esophageal and gastric cases. Interestingly, the samples of columnar-lined mucosa of the esophagus showed aberrant expression of CDX2 and decreased SOX2 despite the absence of intestinal goblet cells (Supplementary Figure S1), and complete GIM showed expression of CDX2 with no detectable SOX2 (Supplementary Figure S2). Our findings show an increase in CDX2 expression and a decrease in SOX2 expression in both normal squamous-BE-EAC and normal gastric gland-GIM-GA progression sequences.

Taken together, our TMA IHC observations hint that BE and pure incomplete GIM are similar and consist of both gastric and intestinal features. But randomly sampled GIM lesions show a mixture between complete and incomplete types as exhibited by a decrease in gastric markers including TFF2, MUC5AC, MUC6, and SOX2. In addition, during esophageal adenocarcinoma progression there is an apparent de-differentiation with loss of both gastric and intestinal characteristics. However, during gastric cancer formation, there is a preferential loss of intestinal markers with maintained expression of gastric markers.

## Discussion

Despite the molecular similarities between EAC and GA (Cancer Genome Atlas Research Network, 2014; Cancer Genome Atlas Research Network, 2017; Zeng and Jin, 2022), there have been few immunohistological studies that have compared these diseases or their precursor metaplastic lesions. The few previous studies directly comparing metaplasias in the stomach and esophagus have focused on the differences between these lesions (El-Zimaity and Graham, 2001; Glickman et al., 2001; Piazuelo et al., 2004) rather than the similarities (Spechler et al., 2017). A key strength of our study has been the focus on similarities *and* differences between BE and GIM by recognizing and highlighting complete and incomplete intestinal metaplasia. In addition, we have also broadened our analysis to include dysplastic and neoplastic lesions of the stomach and esophagus.

To this end, we have analyzed the similarities and differences involved in the formation of EAC and GA using a unique patient tissue microarray of the upper GI tract and a panel of histologic markers including trefoil peptides (TFF2 and TFF3), mucins (MUC2, MUC5AC, and MUC6), and transcription factors (CDX2 and SOX2). Several key findings have emerged from our study. First, we found that most cases of BE in our case series resemble incomplete GIM (Correa et al., 2010; Goldenring and Mills, 2022). Specifically, BE manifests as co-expression of the trefoil factors TTF2 and TTF3, as well as co-expression of MUC2, MUC5AC, and MUC6 (Figures 2–6). In BE, there is also gain of the intestinal transcription factor, CDX2, with concomitant loss of the foregut transcription factor, SOX2 (Figures 7, 8). In contrast, complete GIM is characterized by TFF3 and MUC2 expression without co-expression of TTF2, MUC5AC, or MUC6 (Supplementary Figure S1).

The fact that intestinal metaplasia is heterogenous has been established in the stomach (Correa et al., 2010; Goldenring and Mills, 2022). Using morphology and mucin expression patterns, GIM can be further divided into three distinct types (Filipe et al., 1994). Complete or type I GIM expresses intestinal goblet cell sialomucins reflecting a “pure” intestinal metaplasia (Supplementary Figure S1). Incomplete or type III GIM expresses sulfomucins, and type II (also termed incomplete) expresses both types of mucins reflecting lesions with mixed gastric and intestinal traits (Figure 1D). While these same intestinal metaplasia classifications have not been applied to the esophagus, there has been emerging recognition that BE harbors mixed gastric (specifically more pyloric gland characteristics) and intestinal traits. The mechanism by which this occurs is still unclear; studies have suggested a transdifferentiation process originating from the native squamous epithelium (Huo et al., 2010; Minacapelli et al., 2017), the submucosal glands (Leedham et al., 2008; Owen et al., 2018), or the gastric cardia (Quante et al., 2012; Nowicki-Osuch et al., 2021). We argue that the incomplete intestinal metaplasia seen in BE hints towards the potential origin of these glands initially as normal, oxyntic gastric-type mucosa that undergoes sequential reprogramming events to take on first pyloric, then intestinal characteristics (Goldenring et al., 2010; Jin and Mills, 2018; Goldenring and Mills, 2022).

The clinical implications of these findings are important. Pathologists in the United States diagnose BE largely on the presence of intestinal goblet cells (Naini et al., 2016; Shaheen et al., 2022). However, more “intestinalized” BE lesions have an unclear higher risk of cancer progression (Bhat et al., 2011; Srivastava et al., 2015; Shaheen et al., 2022). In addition, despite a similar risk of cancer development compared to BE (de Vries et al., 2008; Hvid-Jensen et al., 2011; Song et al., 2015), GIM lacks endoscopic cancer screening guidelines in the United States (Gawron et al., 2020; Gupta et al., 2020). Importantly, the risk of gastric cancer development differs between complete and incomplete GIM with incomplete GIM possessing a significantly higher risk of cancer progression (Gonzalez et al., 2016; Gawron et al., 2020). In accordance, emerging clinical practice guidelines have suggested the adoption of surveillance for incomplete GIM (Gawron et al., 2020; Gupta et al., 2020). Thus, the presence of both gastric and intestinal features (i.e., lineage plasticity), may be a driver of cancer progression in the esophagus and stomach. As such, the ability to differentiate intestinal phenotypic differences shown in our study through differential PAS staining patterns, TFF2/TFF3 expression patterns, and MUC2/MUC5AC/MUC6 staining distribution in BE and GIM will be an important clinical management consideration in the future to determine cancer risk.

In our study, we demonstrate a loss of gastric and intestinal markers with the development of EAC and GA. Interestingly, we find that “differentiated” cell features found in the normal stomach and intestines, and in BE and GIM, are often decreased or lost after cancer development. Specifically, during normal esophageal squamous, BE metaplasia, dysplasia, and EAC progression there is a progressive increase in TFF2, TFF3, MUC2, MUC5AC, MUC6, CDX2 with the development of intestinal metaplasia. Then upon further progression to dysplasia and EAC, there is a subsequent decrease in these markers (Figures 2–7). In contrast, during normal gastric gland to GIM progression, there is an increase in intestinal metaplasia markers, TFF3, MUC2, and CDX2, with a decrease in markers normally expressed in gastric cell lineages, TFF2, MUC5AC, and MUC6 (Figures 2–7). With progression from GIM to GA, there is a decrease in TFF2, TFF3, MUC2, and CDX2 with maintained expression of MUC5AC, MUC6, and SOX2 (Figures 2–7).

This retained gastric marker expression in gastric cancer is consistent with certain GAs maintaining a “gastric” differentiation phenotype (Wakatsuki et al., 2008; Boltin and Niv, 2013). While our methods of characterizing EAC and GA have become more sophisticated through the large scale “omics” studies (Cancer Genome Atlas Research Network, 2014; Cancer Genome Atlas Research Network, 2017; Zeng and Jin, 2022), the significance of this differentiation state flux and plasticity has largely been ignored for solid tumors both as a means to develop cancer and a mechanism for cancer treatment resistance (Saenz and Mills, 2018; Jin and Mills, 2019; Zeng and Jin, 2022). In fact, the most persistent, informal histological description of CIN-type tumors in the stomach is misleadingly “intestinal”. While most of these tumors do exhibit intestinal characteristics not normally seen in the stomach, this should not mislead scientists and clinicians into ignoring that the tumors are highly heterogeneous with both intestinal and gastric characteristics that vary from region to region and from patient to patient. Leveraging such plasticity inherent in cancer cells may be an efficacious and safe means to treat cancers in the future (de The, 2018; Enane et al., 2018).

In summary, our study demonstrates the important similarities and differences that occur during the development of intestinal metaplasia and its progression to cancer in the upper GI tract. We show the plasticity that exists during this process as a mix of gastric and intestinal differentiation traits. Our findings also have potential clinical impact for the understanding the molecular bases of EAC and GA. Continued study, especially multi-omic unbiased analyses of the mechanisms underlying the plasticity of the upper GI tract will lead to improved early cancer risk stratification, safer more efficacious oncologic treatments, and perhaps methods to prevent or reverse the development of these metaplasias and dysplasias.

## Data Availability

The original contributions presented in the study are included in the article/Supplementary Materials, further inquiries can be directed to the corresponding authors.

## Supplementary material

SUPPLEMENTARY FIGURE S1 Immunohistochemical analysis of columnar-lined mucosa of the esophagus. **(A)** H&E and **(B)** PAS staining showing morphology of columnar-lined mucosa of the esophagus. Immunohistochemistry showing **(C)** TFF2, **(D)** TFF3, **(E)** MUC2, **(F)** MUC5AC, **(G)** MUC6, **(H)** CDX2, and **(I)** SOX2 staining of columnar-lined mucosa of the esophagus. All scale bars, 500 μm.

SUPPLEMENTARY FIGURE S2 Immunohistochemical analysis of complete gastric intestinal metaplasia. **(A)** H&E and **(B)** PAS staining showing morphology of complete gastric intestinal metaplasia. Immunohistochemistry showing **(C)** TFF2, **(D)** TFF3, **(E)** MUC2, **(F)** MUC5AC, **(G)** MUC6, **(H)** CDX2, and **(I)** SOX2 staining of complete gastric intestinal metaplasia. All scale bars, 500 μm.

SUPPLEMENTARY TABLE S1 The Fisher's exact test *p*-values of IHC marker expression for esophageal cases. P-values were calculated by testing for the difference in the mean IHC scores of the indicated markers. Fisher's exact test was used. *P* < 0.05 is considered statistically significant. Comparisons were between normal and lesion phenotype groups, or 2 lesion phenotype groups (BE-NFD: Barrett’s esophagus negative for dysplasia, BE-LGD: Barrett’s esophagus with low-grade dysplasia, BE-HDG: Barrett’s esophagus with high-grade dysplasia, EAC: esophageal adenocarcinoma).

SUPPLEMENTARY TABLE S2 The Fisher's exact test *p*-values of IHC marker expression for gastric cases. P-values were calculated by testing for the difference in the mean IHC scores of the indicated markers. Fisher's exact test was used. *P* < 0.05 is considered statistically significant. Comparisons were between normal and lesion phenotype groups, or 2 lesion phenotype groups (GIM: gastric intestinal metaplasia, GA: gastric adenocarcinoma).
