# Supplementary material for: Shared features of metaplasia and the development of adenocarcinoma in the stomach and esophagus
Source: Front Cell Dev Biol. 2023 Mar 13;11:1151790. doi: 10.3389/fcell.2023.1151790 (PMC10040611; doi:10.3389/fcell.2023.1151790)
Supplement: Supplementary file 1 [file Table1.DOCX]

**Supplemental Table 1.** **The Fisher's exact test *p*-values of IHC marker expression for esophageal cases**

| Markers | The Fisher's Exact Test *p*-values | | | | | | | | | |
| --- | --- | --- | --- | --- | --- | --- | --- | --- | --- | --- |
|  | Normal vs. BE-NFD | Normal vs. BE-LGD | Normal vs. BE-HGD | Normal vs. EAC | BE-NFD vs. BE-LGD | BE-NFD vs. BE-HGD | BE-NFD vs. EAC | BE-LGD vs. BE-HGD | BE-LGD vs. EAC | BE-HGD vs. EAC |
| TFF2 | 2.843E-13 | 1.2076E-11 | 0.001357 | 0.015428 | 0.111293 | 0.091112 | 1.6436E-10 | 0.179955 | 2.1626E-7 | 0.113653 |
| TFF3 | 9.3177E-11 | 1.0108E-10 | 0.000006 | 6.5682E-9 | 0.567124 | 0.000589 | 0.001118 | 0.002757 | 0.004405 | 0.077808 |
| MUC2 | 1.0301E-9 | 1.4332E-8 | 3.5584E-7 | 0.000088 | 0.790180 | 0.440318 | 0.003587 | 0.640143 | 0.029257 | 0.016688 |
| MUC5AC | 5.3027E-16 | 1.4208E-14 | 1.2559E-7 | 4.5153E-10 | 0.725065 | 0.417198 | 0.000155 | 0.195841 | 0.000858 | 0.050265 |
| MUC6 | 1.5029E-12 | 1.7681E-14 | 1.3003E-8 | 4.5034E-9 | 0.769828 | 0.188491 | 0.002099 | 0.073337 | 0.000038 | 0.065017 |
| CDX2 | 4.8843E-11 | 8.7519E-12 | 2.0448E-8 | 3.0854E-19 | 0.610604 | 0.113536 | 0.679146 | 0.475349 | 0.301170 | 3.0854E-19 |
| SOX2 | 0.002933 | 0.000013 | 0.002431 | 7.1957E-13 | 0.503357 | 0.452545 | 0.004556 | 0.530647 | 0.404451 | 0.158670 |
