# Supplementary figures and images for: Shared features of metaplasia and the development of adenocarcinoma in the stomach and esophagus
Source: Front Cell Dev Biol. 2023 Mar 13;11:1151790. doi: 10.3389/fcell.2023.1151790 (PMC10040611; doi:10.3389/fcell.2023.1151790)

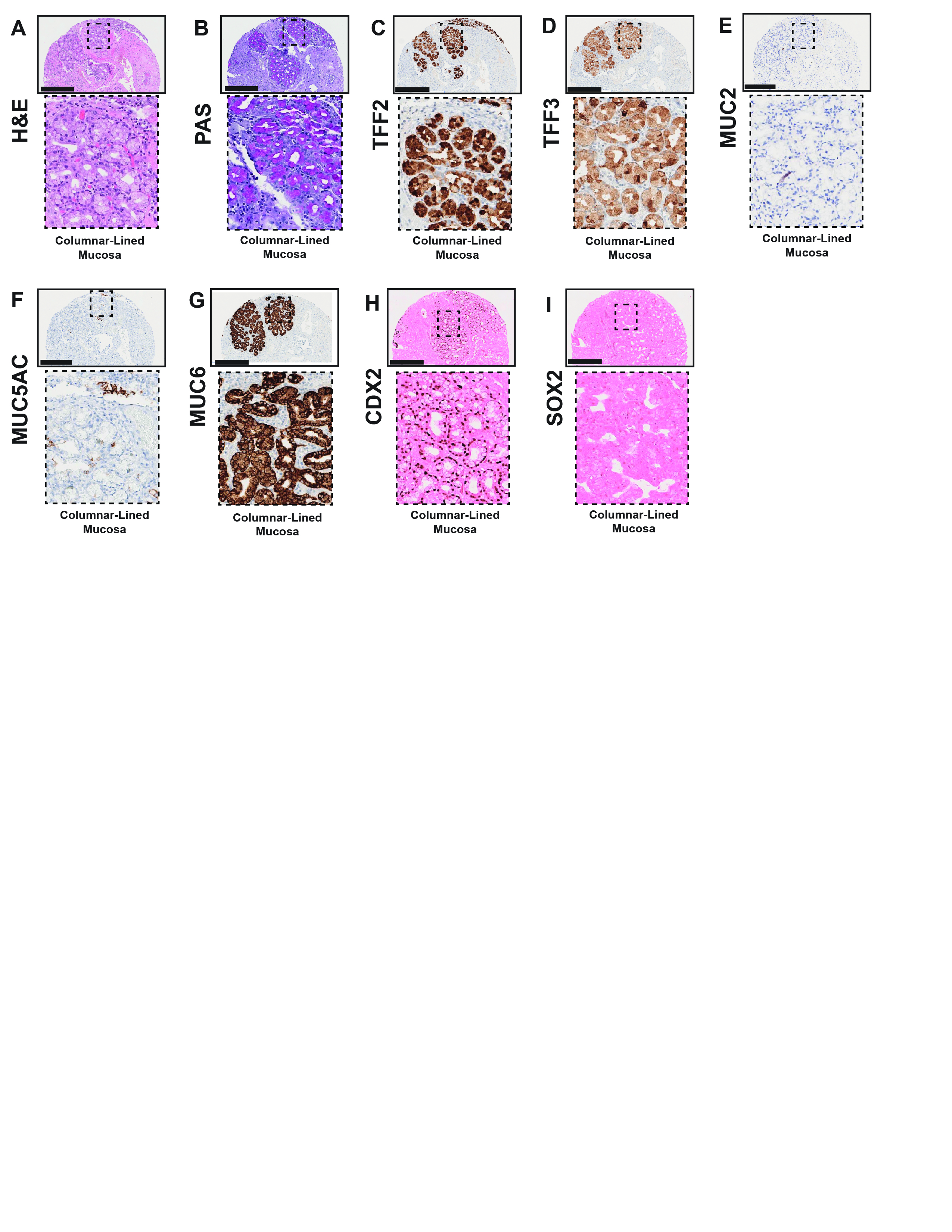

Supplement: Supplementary file 2 [file Image1.JPEG]

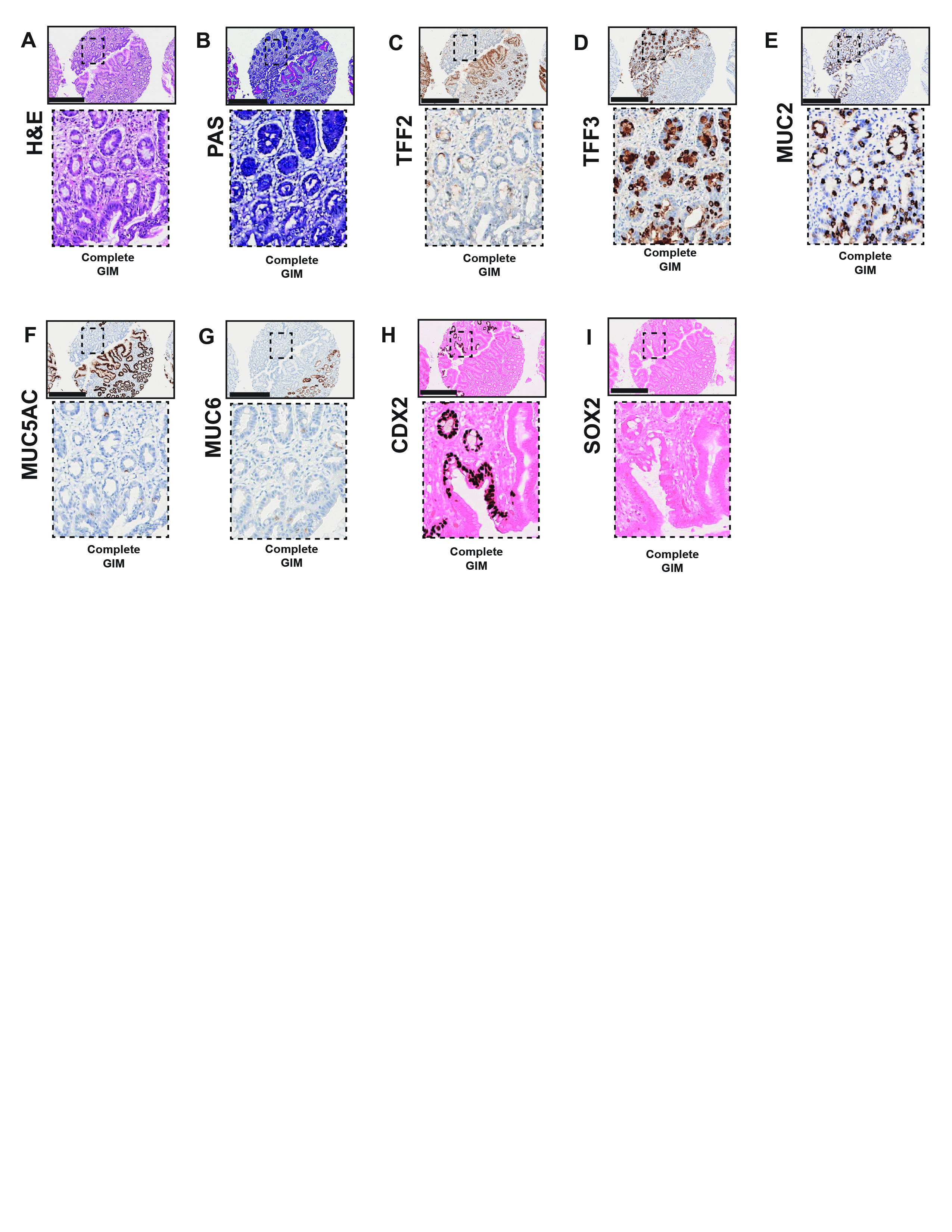

Supplement: Supplementary file 3 [file Image2.JPEG]
