# Supplementary material for: Shared features of metaplasia and the development of adenocarcinoma in the stomach and esophagus
Source: Front Cell Dev Biol. 2023 Mar 13;11:1151790. doi: 10.3389/fcell.2023.1151790 (PMC10040611; doi:10.3389/fcell.2023.1151790)
Supplement: Supplementary file 4 [file Table2.DOCX]

**Supplemental Table 2. The Fisher's exact test *p*-values of IHC marker expression for gastric cases**

| Markers | The Fisher's Exact Test *p*-values | | |
| --- | --- | --- | --- |
|  | Normal vs. GIM | GIM vs. GA | Normal vs. GA |
| TFF2 | 0.000019 | 0.104280 | 1.2203E-14 |
| TFF3 | 2.9836E-17 | 0.000002 | 3.9509E-9 |
| MUC2 | 1.8185E-17 | 0.000234 | 1.3796E-8 |
| MUC5AC | 0.000324 | 1.000000 | 0.000023 |
| MUC6 | 1.6333E-10 | 0.649867 | 4.8215E-13 |
| CDX2 | 1.1607E-16 | 0.131462 | 2.6122E-17 |
| SOX2 | 0.000002 | 0.540825 | 0.000002 |
